# Determinants of use of the fixed dose combination emtricitabine/rilpivirine/tenofovir (Eviplera) in HIV-infected persons receiving care in Italy

**DOI:** 10.7448/IAS.17.4.19775

**Published:** 2014-11-02

**Authors:** Alessandro Cozzi-Lepri, Sergio Lo Caputo, Franco Maggiolo, Andrea Antinori, Adriana Ammassari, Giulia Marchetti, Claudio Mastroianni, Andrea Gori, Giovanni Di Perri, Gioacchino Angarano, Alessia Carbone, Antonella d'Arminio Monforte, Study, on behalf of the ICONA Foundation

**Affiliations:** 1 Infection and Population Health, University College London, London, Italy; 2 Infectious Diseases Clinic, S. M. Annunziata Hospital, Firenze, Italy; 3 Infectious Diseases, Ospedali Riuniti Bergamo, Bergamo, Italy; 4 Infectious Diseases, INMI Spallanzani Hospital, Rome, Italy; 5 Health Sciences, San Paolo Hospital, Milan, Italy; 6 Infectious Disease, La Sapienza University Rome, Rome, Italy; 7 Infectious Diseases, San Gerardo University Monza, Monza, Italy; 8 Infectious Diseases, University of Turin, Turin, Italy; 9 Biomedical Sciences, University of Bari, Bari, Italy; 0 Infectious Diseases, San Raffaele Hospital, Milan, Italy; 1 Health Sciences, University of Milan, Milan, Italy

## Abstract

**Introduction:**

Emtricitabine/rilpivirine/tenofovir (EVP) is a fixed-dose combination of antiretrovirals (ARV) approved by the European Medicines Agency in November 2011 and introduced in Italy in February 2013. It is a once-a-day single tablet and is licensed in Europe for use only in ARV-naïve patients with a viral load (VL) ≤100,000 copies/mL.

**Objective:**

To identify factors that may be associated with the use of EVP as first-line regimen in HIV-infected individuals starting cART from ARV-naïve in Italy.

**Methods:**

Clinical sites in ICONA Foundation Study in which ≥1 person had started EVP were selected for this analysis. From these we included all patients who started an EVP-based cART regimen as well as those starting other cART regimens after the date of introduction of EVP at the site (after February 2013 in any case) and with a VL ≤100,000 copies/mL from ARV-naïve. Characteristics at the time of starting cART were compared using chi-square test and unadjusted and adjusted logistic regression analysis. Factors investigated included: gender, mode of HIV transmission, time from HIV diagnosis, CD4 count, nation of birth, AIDS, HCV-status, age, CD8 count, VL, diabetes, smoking, total and HDL cholesterol, eGFR, blood glucose, level of education and employment and site location. Factors showing unadjusted associations with a p-value of 10% or smaller, were retained in the multivariable model.

**Results:**

We identified 183 patients starting EVP and 173 starting the control regimen from 23 sites. The number of patients starting EVP included at each site ranged from 1 to 12 and the number of those starting the control regimen was similar. The most frequently used drugs in the concurrent group were: TDF (75%), FTC (74%), DRV (39%), ATV/r (26%), LPV/r (9%), EFV (13%) and RAL (14%). In univariable analysis, there were differences in median CD4 count (390 cells/mm^3^ in EVP versus 348 in controls, p=0.002), time from HIV diagnosis to starting cART (11 versus 3 months, p=0.001) and prevalence of students (6% versus 3%, p=0.07). No differences were observed for all other factors examined. The table shows estimates of the odds ratios (OR) for factors included in the multivariable model.

**Conclusions:**

CD4 count was higher in EVP-treated patients compared to controls. Guidelines suggest avoiding initiation of EVP in presence of high VL, possibly explaining this residual difference in CD4. There was also a tendency to prescribe EVP to people with perceived lower adherence or hesitant to start or perhaps with a slow progressing disease.

**Table 1:** Odds ratios of starting Eviplera from fitting a logistic regression model

	Odds ratios of starting eviplera			
	
Characteristic	Unadjusted OR (95% CI)	p-value	Adjusted OR (95% CI)	p-value
CD4 count, cells/mmc (per 100 cells higher)	1.15 (1.03, 1.28)	0.014	1.17 (1.04, 1.32)	0.008
Time from HIV diagnosis to date of starting cART(per year longer)	1.11 (1.03, 1.18)	0.004	1.10 (1.03, 1.18)	0.008
Employment, n (%)				
Unemployed	1.00		1.00	
Employed	1.69 (0.80, 3.55)	0.166	1.78 (0.79, 4.01)	0.162
Self-Employed	1.20 (0.50, 2.84)	0.686	1.25 (0.50, 3.14)	0.640
Occasional	1.71 (0.48, 6.11)	0.408	2.18 (0.58, 8.27)	0.251
Student	3.23 (0.93, 11.19)	0.065	3.49 (0.93, 13.08)	0.64
Retired/Invalid/Housewife	1.05 (0.28, 3.93)	0.945	0.65 (0.15, 2.71)	0.551
Other/unknown	1.72 (0.80, 3.68)	0.165	1.85 (0.79, 4.31)	0.155

